# Multiple virus sorting based on aptamer-modified microspheres in a TSAW device

**DOI:** 10.1038/s41378-023-00523-1

**Published:** 2023-05-17

**Authors:** Xianglian Liu, Xuan Chen, Yangchao Dong, Chuanyu Zhang, Xiaoli Qu, Yingfeng Lei, Zhuangde Jiang, Xueyong Wei

**Affiliations:** 1 State Key Laboratory for Manufacturing Systems Engineering, Xi’an Jiaotong University, Xi’an, 710049 China; 2 Department of Microbiology, the Fourth Military Medical University, Xi’an, 710032 China

**Keywords:** Microfluidics, Biosensors

## Abstract

Due to the overlapping epidemiology and clinical manifestations of flaviviruses, differential diagnosis of these viral diseases is complicated, and the results are unreliable. There is perpetual demand for a simplified, sensitive, rapid and inexpensive assay with less cross-reactivity. The ability to sort distinct virus particles from a mixture of biological samples is crucial for improving the sensitivity of diagnoses. Therefore, we developed a sorting system for the subsequent differential diagnosis of dengue and tick-borne encephalitis in the early stage. We employed aptamer-modified polystyrene (PS) microspheres with different diameters to specifically capture dengue virus (DENV) and tick-borne encephalitis virus (TBEV), and utilized a traveling surface acoustic wave (TSAW) device to accomplish microsphere sorting according to particle size. The captured viruses were then characterized by laser scanning confocal microscopy (LSCM), field emission scanning electron microscopy (FE-SEM) and reverse transcription-polymerase chain reaction (RT‒PCR). The characterization results indicated that the acoustic sorting process was effective and damage-free for subsequent analysis. Furthermore, the strategy can be utilized for sample pretreatment in the differential diagnosis of viral diseases.

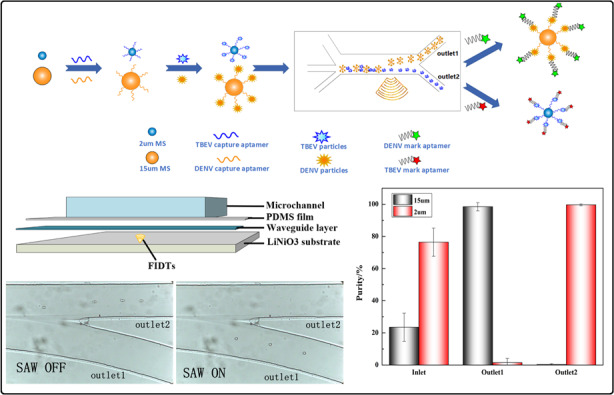

## Introduction

Viral infections are a grave threat to public health as well as the global economy due to their high pathogenicity for humans and animals. Viral infections mostly occur through contaminated water, food, bodily fluids, and even air, spread rapidly and result in the death of humans and animals worldwide^1^. Flaviviruses, the leading cause of arthropod-borne diseases in humans, are single-stranded RNA, enveloped viruses belonging to the Flaviviridae family. Flaviviruses include 53 recognized virus species, of which 40 species are known to cause disease in humans. The most well-known members include dengue virus (DENV), Zika virus (ZIKV), Japanese encephalitis virus (JEV), West Nile virus (WNV), yellow fever virus (YFV), tick-borne encephalitis virus (TBEV) and Langat virus (LGTV)^2^. Among these flaviviruses, DENV is one of the most important mosquito-borne viruses, and TBEV is among the most common tick-transmitted pathogens. There are many flu-like initial symptoms, including fever, fatigue, general malaise, headache and muscular pain, at the early stage of flavivirus infection^3^. Therefore, differential diagnosis of dengue and tick-borne encephalitis (TBE) in the early stage based on clinical symptoms alone is unreliable^4^. Inaccurate diagnosis may cause unnecessary hospital admission and in turn increase the burden on health care systems. Conventional laboratory diagnostic assays have long been used as tools for detecting DENV in clinical practice. Nonetheless, these detection methods, e.g., virus isolation–based molecular assay methods, are time-consuming, difficult, and require skilled technicians and central laboratory facilities. The major problem with serological methods for DENV detection is their strong predisposition to cross-react with antibodies, which are produced in response to other simultaneous and previous flavivirus infections (e.g., JEV, YFV, and TBEV)^5^. Therefore, there is a perpetual demand for a simplified, sensitive, rapid and inexpensive assay with less cross-reactivity. To meet the demand, the ability to sort distinct virus particles from a mixture of biological samples is crucial for improving the sensitivity of the test.

Due to the nanometer size of virus particles, it is difficult to sort distinct virus particles from biological samples directly. To sort the specific virus, aptamer-modified solid substrates (e.g., microspheres and magnetic beads) were developed to capture different viruses. Aptamers are RNA or ssDNA molecules of approximately 20 to 80 bases with a specific conformational affinity toward target molecules, including proteins, small molecules, metal ions, bacterial cells, viruses, cancer cells, and even tissues^6^. Similar to the conformational recognition between antibodies and antigens, aptamers bind to their cognate targets with high specificity and affinity through various types of interactions, such as van der Waals forces, hydrogen bonds, electrostatic interactions and bonds between complementary bases^7–9^. Due to their striking advantages over traditional antibodies, such as ease of selection, low cost of synthesis, high stability-specificity-sensitivity, and potential for modifications and tagging, aptamers are considered potential alternatives to monoclonal antibodies in many fields, including diagnostics, therapeutics, cell imaging, drug delivery, selective chromatography, biosensing, and biomarker discovery^10^. The characteristic properties of the aptamers previously mentioned make them valuable tools for research in viral diagnosis and therapy against viral infection as well. Many previous studies have used aptamers to capture and detect viruses such as human papillomavirus (HPV)^11^, hepatitis B virus (HBV)^12^, hepatitis C virus (HCV)^13,14^, norovirus (NoV)^15–17^, ZIKV^18,19^, and DENV^20,21^. Fletcher et al.^20^ developed an aptamer-based biosensor using DNA aptamers for the DENV genome, generating fluorescence to confirm the presence of dengue sequences. Basso et al.^21^ reported an aptasensor composed of γ-Fe_2_O_3_(SAMN) nanoparticles and modified with gold nanoparticles (AuNPs) conjugated to aptamers that can specifically bind to four dengue serotypes.

When sorting micro- or nanoparticles, microfluidic devices exhibit several advantages, including the capability of being integrated into various drive systems, including acoustic^22,23^, electrical^24^, magnetic^25^, optical^26^, and mechanical systems^27^. Among the various techniques, acoustofluidics, the integration of acoustics and microfluidics, can separate particles depending on their different physical properties, such as density, size, and compressibility. Acoustofluidic separation offers a highly controllable, label-free, contactless, damage-free and biocompatible approach that relies on the differential effect of acoustic streaming and radiation forces acting on particles suspended in a liquid^28–30^. It has increasingly been applied in the separation of different biological targets from complex fluids, such as proteins^31,32^, circulating tumor cells (CTCs)^33^, viruses^34^ and small extracellular vehicles (EVs)^35–41^. Sung et al.^31^ simultaneously separated proteins of three kinds (thrombin, immunoglobulin E, and mCardinal2) via TSAW-driven ARF in the proposed acoustofluidic device, which can be used as a promising tool for various protein-based clinical diagnostic assays. Wu et al.^33^ introduced a high-throughput, acoustic-based CTC-separation device that used tilted-angle standing surface acoustic waves for ex vivo CTC growth studies and CTC phenotypic studies. They further achieved the successful isolation and phenotypic characterization of CTCs from clinical patient samples with metastatic prostate cancer. Later, the same group reported a standing surface acoustic wave (SSAW) device to separate EVs and lipoproteins based on their acoustic properties, further expanding the capability of acoustofluidic separation technology for separating submicron-scale objects^35^. The study of Ku et al.^37^ proposed a noncontact platform that makes use of ultrasonic wave scattering between micrometer-sized seeding particles and nanoparticles of interest in a resonant cavity for EV enrichment. The platform named acoustic trapping can be used to isolate EVs, including microvesicles and exosome-size vesicles, from cell culture conditioned media, human urine, and plasma samples. Most studies mentioned above have focused on separating one special biological target from complex fluids, but few studies have been concerned with sorting multiple different targets from samples.

In this study, we developed an efficient multiple virus sorting method based on aptamer-modified microspheres in a traveling surface acoustic wave (TSAW) device. Here, we employed aptamer-modified polystyrene (PS) microspheres with different diameters to specifically capture DENV and TBEV and utilized a TSAW device to accomplish microsphere sorting according to particle size. The sorted viruses were characterized by laser scanning confocal microscopy (LSCM), field emission scanning electron microscopy (FE-SEM) and reverse transcription-polymerase chain reaction (RT‒PCR) to confirm that the strategy was suitable for subsequent tests.

## Materials and Methods

### Experimental materials and reagents

Carboxy polystyrene (PS) microspheres were purchased from Unibead Scientific Co. Ltd. (Tianjin, China). N-hydroxysulfosuccinimide (Sulfo-NHS) and 1-ethyl-3-(3-dimethylaminopropyl) carbodiimide hydrochloride (EDC) were purchased from Thermo. Phosphate buffer solution (PBS) (pH 7.4), trihydroxymethyl aminomethane-hydrochloric acid (Tris-HCl) and 2-(N-morpholino) ethanesulfonic acid hydrate (MES) buffer (0.05 mol, pH 6.0) were purchased from Leagene Biotechnology (Beijing, China). Dengue virus (DENV), tick-borne encephalitis virus-like particles (TBEV), Japanese encephalitis virus (JEV), and coxsackie virus B3 (CVB3) were obtained from the Fourth Military Medical University. Bovine serum albumin (BSA) and fetal bovine serum (FBS) were purchased from Sangon Biotechnology (Shanghai, China).

### Fabrication of the TSAW device

The main structure parameters of IDTs in the TSAW device design we need to consider are the aperture, the width and the pitch of each electrode finger, and the number of finger pairs (N). Normally, the size of the width and pitch of each linear electrode finger is designed with the same value in microfluidic devices. The focused interdigital transducers (FIDTs) with arc electrodes in our microfluidic device contain 15 pairs of focused electrodes, and the acoustic aperture is 1 mm. The designed width and pitch of each electrode is 20 μm, corresponding to a TSAW wavelength of 80 μm. The degree of the arc of the FIDTs is set to 60°, and the induced SAW is considered to have smaller energy attenuation, higher focused energy and longer propagating distance^42^. The width and height of the polydimethylsiloxane (PDMS) microchannel are 300 μm and 80 μm, respectively. To fabricate the acoustofluidic device, the photoresist (EPG535, Everlight Chemical, Taiwan, China) was patterned on the transparent LiNbO_3_ substrate by standard lithography, then metal layers (Cr/Au, 50/200 nm) were deposited on the substrate by electron beam evaporation (TF500, HHV, England). Finally, lift-off technology was used to obtain the target IDTs. The PDMS microchannel was fabricated by standard soft lithography technology. The microchannel mold was fabricated on the silicon wafer using SU8 photoresist. PDMS was mixed with the curing agent at a ratio of 10:1 and poured into the microchannel mold. After curing for 4 h at 85 °C, the PDMS microchannel was peeled off from the mold and cut into an appropriate size. The PDMS microchannel was bonded with a thin PDMS film (50 μm thick) after oxygen plasma treatment. Finally, to fabricate the TSAW device, the PDMS microchannel was aligned on the IDTs with oil as a waveguide layer. This type of prebonded microchannel permits easier replacement of the microchannel and the IDTs and greatly reduces the fabrication cost of the acoustofluidics device. The structure and image of the TSAW device are shown in Fig. 2a.

### Aptamers and primers

Aptamers used for virus capture and primers applied in the RT‒PCR procedure were synthesized by Sangon Biotechnology (Shanghai, China). The sequences are listed in Table S1. The hairpin structure of the aptamers was analyzed by the Mfold web platform^43^.

### Aptamer modification of microspheres

First, a 50 μL solution of carboxy-coated PS microspheres (250 mg/10 mL, 15 μm in diameter) was washed three times with PBS buffer to remove the storage solvent completely and resuspended in 500 μL of MES buffer (0.05 mol, pH 6.0). Then, 10 mg of EDC and 10 mg of Sulfo-NHS were added to the above MES buffer and reacted for 15 min at room temperature. Then, the PS microspheres were washed twice quickly with PBS buffer and resuspended in 500 μL of MES buffer (0.05 mol, pH 6.0). Here, the washing procedure can remove the excess reagent and is useful for avoiding the influence of hydrolysis or excessive EDC reaction on aptamer modification. A 10 μL solution of DENV capture aptamer (5 µM) was added into the above solution and reacted at room temperature for 2 h with shaking at 180 r/min. Finally, the aptamer-modified PS microspheres (PS15-A) were blocked with 300 μl of 3% BSA for 30 min, suspended in 500 μL of PBS (pH 7.4), and finally stored at 4 °C for further use. The blocking procedure is beneficial to reduce nonspecific adsorption and binding on the microsphere surface. In accordance with the aforementioned method, the TBEV capture aptamer was conjugated on the 2 μm PS microspheres to prepare the complexes (PS2-A). The conjugation between the aptamer and the PS microspheres was verified by Fourier transform infrared spectroscopy (FTIR) on a Nicolet iS10 (Thermo Fisher, America).

### Sorting target viruses

**Fig. 1:**
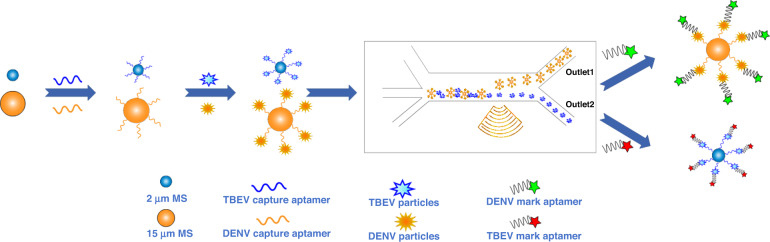
Schematic of the process for acoustofluidic sorting of two different viruses. Viruses are specifically captured by aptamer-modified PS microspheres with different sizes and are then sorted into different outlets by the function of surface acoustic wave, to achieve high purity of each type of targets

The schematic workflow of the acoustofluidic sorting of DENV and TBEV particles is shown in Fig. 1. First, two different aptamers targeting DENV and TBEV were modified on PS microspheres of different sizes, 15 μm and 2 μm in diameter, respectively. Then, the aforementioned 500 μL solution of PS15-A and PS2-A was centrifuged and added to 500 μl sample solution containing DENV and TBEV at a certain concentration and mixed thoroughly for 1 h with shaking at 180 r/min. The sample solution was prepared by mixing virus particles with PBS or FBS to simulate the physiological environment.

**Fig. 2:**
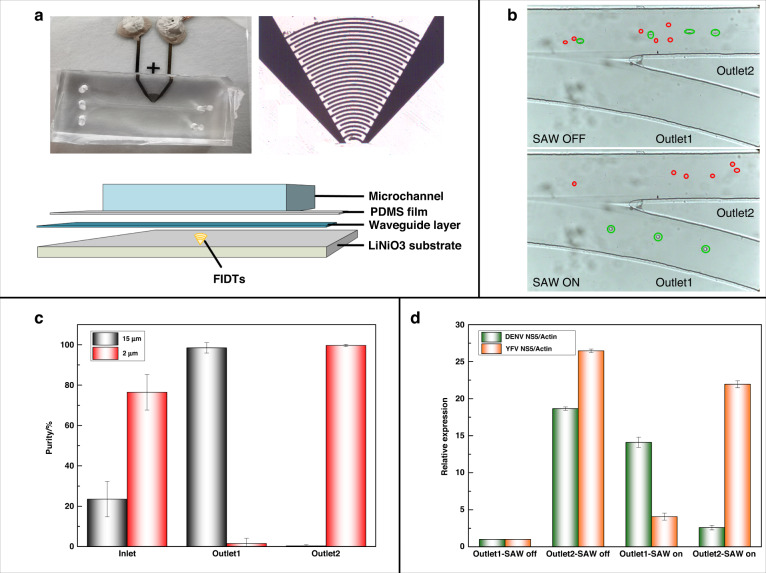
Acoustofluidic sorting of DENV and TBEV. **a** Structure and image of the TSAW device. **b** The sorting outlets when SAW switched off/on (15 μm PS microspheres in green circles and 2 μm PS microspheres in red circles). **c** The purity of the 15 μm PS microspheres and 2 μm PS microspheres at the inlet, outlet 1 and outlet 2. **d** The relative genetic expression of the virus on the sorted microspheres

Second, the incubated samples were injected into the microchannel of the TSAW device (shown in Fig. 2a) for the sorting procedure. Acoustofluidic devices based on surface acoustic waves (SAWs) have been demonstrated to be effective for fluid mixing, atomization, particle manipulation, and sorting^28^. Here, we used a typical TSAW on 128° rotated Y-cut lithium niobate substrates generated by a single IDT oriented at a certain angle with respect to the X-direction of the crystal. The TSAW propagated toward the PDMS microchannel and radiated into the fluid at a Rayleigh angle θ_R_, and θ_R_ was approximately 22°, as calculated by the following Eq. (1):

1 $$\theta _{\mathrm{R}} = {\mathrm{sin}}^{ - 1}{\mathrm{c}}_{\mathrm{f}}{/}{\mathrm{c}}_{\mathrm{s}}$$

c_f_ and c_s_ are the speed of sound in the fluid and on the LiNbO_3_ substrate, respectively, with c_f_ = 1495 m/s and c_s_ = 3978 m/s. The suspended particles in the fluid were pushed laterally away from the acoustic source and approached the roof of the microchannel due to the acoustic radiation force consistent with the Rayleigh angle. In the TSAW field, the dimensionless factor κ was defined to evaluate the acoustic radiation force acting on microparticles indirectly. The value of κ was determined by the diameter of the particles and acoustic wave frequency, as shown in Eq. (2)^44^.

2 $$\kappa = \pi {\mathrm{d}}{{\mathrm{f}/{\mathrm{c}}_{\mathrm{f}}}}$$

where d is the diameter of the polystyrene microsphere, f is the design frequency of surface acoustic waves, and c_f_ is the acoustic speed in the fluid.

Due to spherical isotropic scattering, the acoustic radiation force acting on microparticles disappears when κ is less than 1. When κ is greater than 1, backscattering is dominant, and net momentum is transferred to the particles. The value of κ is determined by the diameter of microparticles (d) and acoustic wave frequency (f). Here, we employed 2 μm and 15 μm PS microspheres to sort different viruses, and the values of κ were 0.21 to 2 μm and 1.57 to 15 μm, respectively, under the 50 MHz TSAW. This means that only particles with a size of 15 μm could be affected by the radiation force and induce a differential streamline for particles under TSAW. The PS microsphere-Aptamer-DENV (PS15-A-DENV) and PS microsphere-Aptamer-TBEV (PS2-A-TBEV) complexes were sorted from the mixed virus solution in the PDMS microchannel by TSAW. Briefly, the mixed sample solution was injected into the center inlet of the PDMS microchannel. Two PBS sheath flows were injected into the adjacent inlets, and the sample solution was forced into a narrow and straight line. The flow rate in the microchannel was controlled within 2000-2800 μl per hour by regulating the inlet pressure. To achieve high separation efficiency, the limit was calculated as 2800 μl per hour in the situation, in which the maximum tolerable power input was set on the IDTs. When the IDTs were turned on with a frequency of 46 MHz and an input power of 8 W, the PS15-A-DENV complexes were driven into the PBS stream and collected in outlet 1. The rest of the sample solution containing PS2-A-TBEV was collected in outlet 2.

### Characterizing the captured viruses

The collected PS15-A-DENV complexes in outlet 1 were labeled with the DENV marker aptamer conjugated with fluorophore 6-FAM, while the collected PS2-A-TBEV composites in outlet 2 were labeled with the TBEV mark aptamer conjugated with fluorophore Texas Red. Then, the labeled complexes were observed by laser scanning confocal microscopy (LSCM) and FS5 spectrofluorometry (Edinburgh Instruments). At the same time, samples containing Japanese encephalitis virus (JEV) and Coxsachie virus B3 (CVB3) were used as parallel controls to ensure the specific recognition ability of the DENV capture aptamer and the TBEV capture aptamer.

The surface morphologies of the PS15-A-DENV complexes and PS2-A-TBEV complexes were obtained by field emission scanning electron microscopy (FE-SEM, SU-8010, Japan). The PS particles were dispersed on the cooper mesh and observed by FE-SEM. Reverse transcription-polymerase chain reaction (RT‒PCR) was employed to detect DENV NS5 and YFV NS5 genetic expression to further confirm the successful capture of DENV and TBEV. Briefly, the viruses captured by the PS-A were lysed to extract the RNAs, and then RNAs were converted into their complementary DNA (cDNA) sequences by reverse transcriptase, followed by amplification of the newly synthesized cDNA by standard polymerase chain reaction (PCR) procedures. The PCR product was detected by real-time fluorescent quantitative PCR on a CFX 96 Touch (Bio-Rad, America), and the results were analyzed by Bio-Rad CFX Manager3.

## Results and Discussion

### Structure of the aptamer-modified microspheres

The secondary structure of the aptamers was analyzed by the Mfold web platform and is shown in Fig. S1. The Mfold web platform is the Mfold web server for nucleic acid folding and hybridization prediction^43^. All the selected aptamers have classic hairpin structures that help the aptamers recognize and capture targets. The DENV capture aptamer and mark aptamer were both specific for the four dengue serotypes. Their efficacy and specificity were verified in the work of Chen et al.^45^ The TBEV capture aptamer and mark aptamer can bind TBEV effectively and specifically^46^.

The conjugation between the aptamer and the PS microspheres was verified by Fourier transform infrared spectroscopy (FTIR)^21,47^. As shown in Fig. S2, there were the characteristic peaks of 1028 cm^−1^ and 1068 cm^−1^ of the benzene ring in the polystyrene and the characteristic peaks of 2849 cm^−1^ and 2923 cm^−1^ of the -CH_2_- group in the PS microspheres. Compared to the control PS microspheres, the microsphere samples coupled with the aptamer produced an additional 1420 cm^−1^ characteristic peak of amide bond -C–N- stretching vibration, 1600 cm^−1^ characteristic peak of -NH- flexural vibration, and 1715 cm^−1^ characteristic peak of C and G bases before and after virus capture. The amide -C–N- bond was formed between the aptamer 5′ end conjugated -NH_2_ group and the -COOH group modified on the PS microspheres. A slight difference in the FTIR spectrograms was obtained after virus capture. This may be because the main components of virus, RNA, and proteins share most groups with the BSA and aptamer on the modified PS microspheres.

### Sorting of DENV and TBEV by the TSAW device

The PS microsphere-Aptamer-DENV (PS15-A-DENV) and PS microsphere-Aptamer-TBEV (PS2-A-TBEV) complexes were sorted from the mixed virus solution in the PDMS microchannel by a TSAW device. As shown in Fig. 2b, when the TSAW was switched off, both the 2 μm and 15 μm PS microspheres flowed along the original streamline and into outlet 2. When the TSAW was switched on, the 15 μm PS microspheres deviated from their original streamline and flowed into outlet 1, and the 2 μm PS microspheres moved along the original streamline and into outlet 2. The purities of 15 μm and 2 μm PS microspheres in the samples were obtained by counting microspheres using a hemocytometer and are shown in Fig. 2c. The sample we injected contained 23.53 ± 8.77% 15 μm PS microspheres. After sorting, we obtained samples with a 15 μm PS microsphere purity of 98.46 ± 2.56% from outlet 1 and a 2 μm PS microsphere purity of 99.67 ± 0.57% from outlet 2. As the target viruses have been completely mixed with the specifically modified microspheres by a thermostatic shaker before the sorting process, the separation efficiency of different viruses only depends on the corresponding efficiency of the PS microspheres. The relative genetic expression of virus on microspheres collected from outlet 1 and outlet 2 was detected by RT‒PCR with actin as the housekeeper gene and calculated with the samples collected from outlet 1 when the SAW switched off as the control group. As shown in Fig. 2d, when the SAW switched on, the sample collected from outlet 1 obtained definitely higher expression of DENV NS5 than YFV NS5, whereas higher YFV NS5 expression was measured in the samples from outlet 2. The polarization of the gene expression in the sorted samples indicated that the purification of viruses was successful.

### Characterization of the captured virus and subsequent tests

**Fig. 3:**
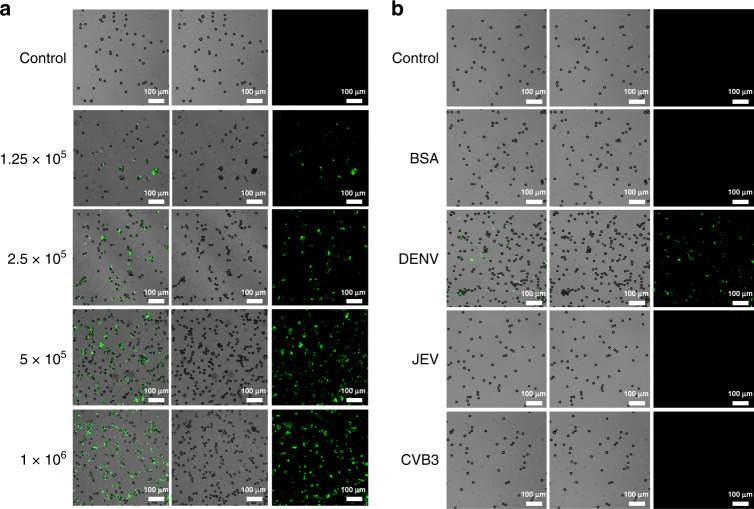
Qualitative detection of viruses captured by 15 μm PS microspheres. **a** Confocal microscopy fluorescence photographs of the complexes of PS15-A isolated from samples with different virus concentrations at 488 nm. **b** Confocal microscopy fluorescence photographs of the complexes of PS15-A isolated from different samples at 488 nm. PBS samples were used as the control group

**Fig. 4:**
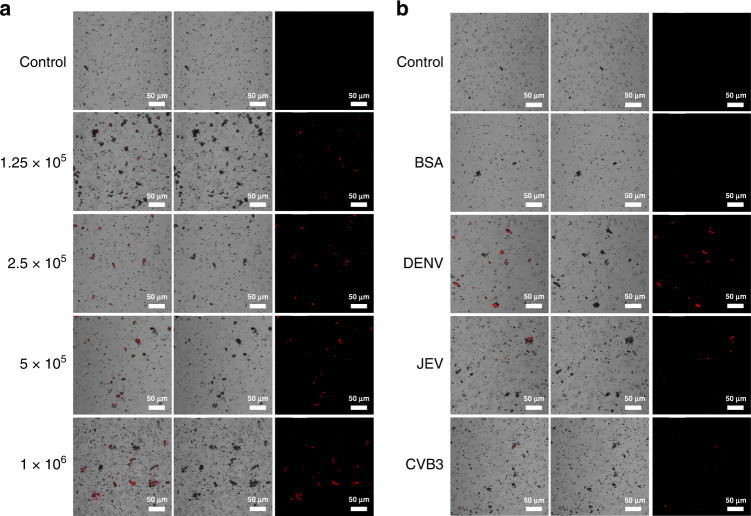
Qualitative detection of viruses captured by 2 μm PS microspheres. **a** Confocal microscopy fluorescence photographs of the complexes of PS2-A isolated from samples with different virus concentrations at 561 nm. **b** Confocal microscopy fluorescence photographs of the complexes of PS2-A isolated from different samples at 561 nm. PBS samples were used as the control group

The PS microspheres collected from outlet 1 and outlet 2 were labeled with 6-FAM coupled aptamer (DENV mark aptamer) and Texas Red coupled aptamer (TBEV mark aptamer), respectively. Subsequently, the labeled samples were observed by laser scanning confocal microscopy (LSCM) excited at 488 nm and 561 nm. The obtained confocal microscopy photographs are shown in Fig. 3 and Fig. 4. Samples with different DENV and TBEV concentrations were handled to determine the relationship between fluorescence intensity and virus concentration. According to Fig. 3a and Fig. 4a, the observed fluorescence signal was heightened along with the virus concentration in the samples, while few fluorescence signals were detected in the control group. To verify the specific recognition ability, JEV and CVB3 were used as parallel groups for DENV capture, while DENV and JEV were used for TBEV capture. As shown in Fig. 3b, the DENV group emitted evident green fluorescence, and the JEV and CVB3 groups emitted few fluorescence signals, as did the control group and BSA group. According to the results shown in Fig. 4b, the samples emitted less fluorescence than that of the TBEV group. These results indicated that PS15-A could capture DENV, while PS2-A could capture TBEV specifically, and the fluorescence signal was positively correlated with the virus concentration.

**Fig. 5:**
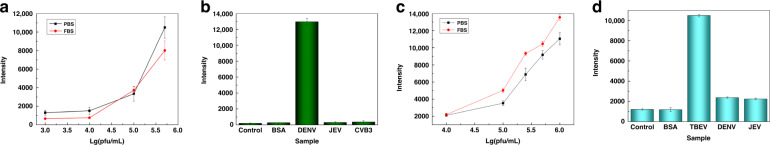
Fluorescence intensity of samples containing different aspecific viruses. **a**, **b** Fluorescence intensity of the complexes of PS15-A isolated from different samples at 488 nm. **c**, **d** Fluorescence intensity of the complexes of PS2-A isolated from different samples at 561 nm. PBS samples were used as the control group

However, LSCM is regularly used for qualitative detection. Due to the smaller scale and lower distinguishability of 2 μm PS microspheres, red fluorescence was more difficult to detect by LSCM than green fluorescence. To achieve quantitative detection of fluorescence, we employed an FS5 spectrofluorometer to obtain the fluorescence intensity of the samples in FBS and PBS to simulate the physiological environment. In this work, we adopted fluorescence intensity for semiquantitative detection of different viruses. As shown in Fig. 5b and d, the detected fluorescence intensity of samples containing different aspecific viruses was consistent with the results obtained by LSCM. The samples prepared by FBS were employed to simulate a more complex physiological environment than that of PBS. The complex components in FBS, such as proteins, peptides, lipids, growth factors, and hormones, may impact the fluorescence intensity of the microspheres. The results shown in Fig. 5a, c indicated that FBS had a slight impact on the detection of fluorescence, with a reduction for PS15-A-DENV and an increase for PS2-A-TBEV. In consideration of the impact of the liquid environment, we need to establish a standard curve before we employ fluorescence intensity to quantify the virus concentration.

**Fig. 6:**
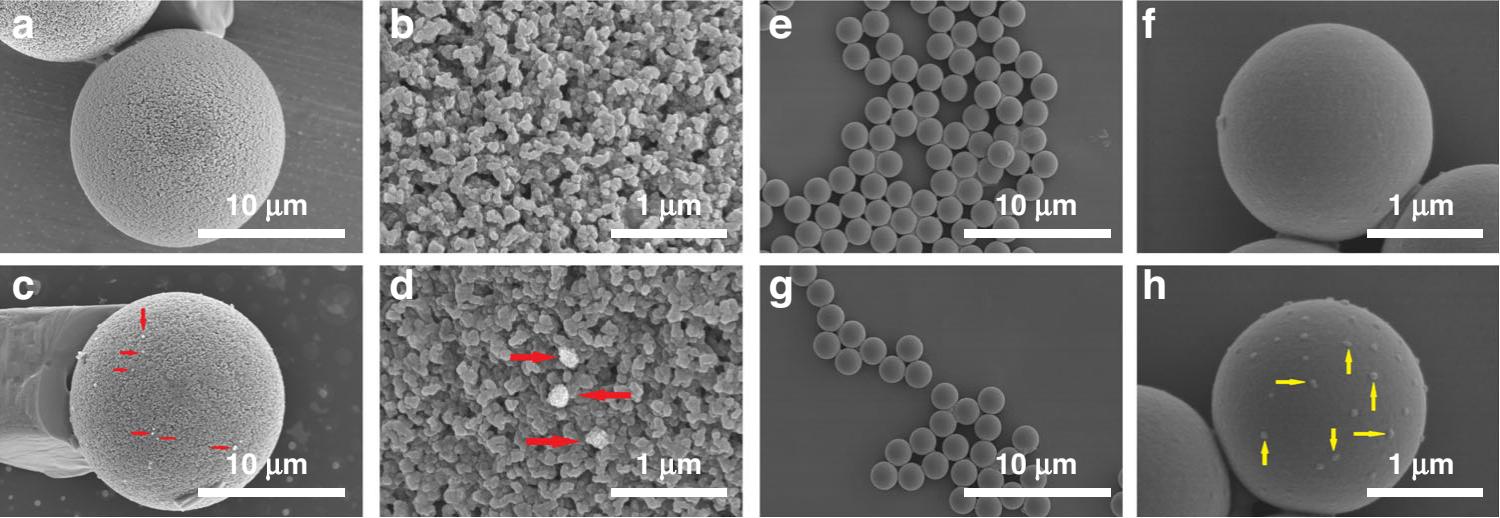
SEM photographs of the surface morphology of the PS microspheres. **a**, **b** PS15 microspheres. **c**, **d** PS15-A-DENV complexes. **e**, **f** PS2 microspheres. **g**, **h** PS2-A-TBEV complexes. Red arrows indicate DENV particle adhesion on the PS15 microsphere surface, yellow arrows indicate TBEV particle adhesion on the PS2 microsphere surface

The SEM photographs of PS15-A-DENV and PS2-A-TBEV complexes were obtained by FE-SEM to visually confirm the capture of virus and are shown in Fig. 6. The surfaces of the PS microspheres were smooth and clean; in contrast, visible virus particle adhesion was observed on the surfaces of PS15-A-DENV and PS2-A-TBEV. In Fig. 6d and 6h, we observed that the DENV particles (red arrows) were larger than the TBEV particles (yellow arrows). The DENV particles were approximately 100 nm in diameter with a spiky surface^48^, while the TBEV particles were approximately 50 nm in diameter with a smooth surface^49^.

**Fig. 7:**
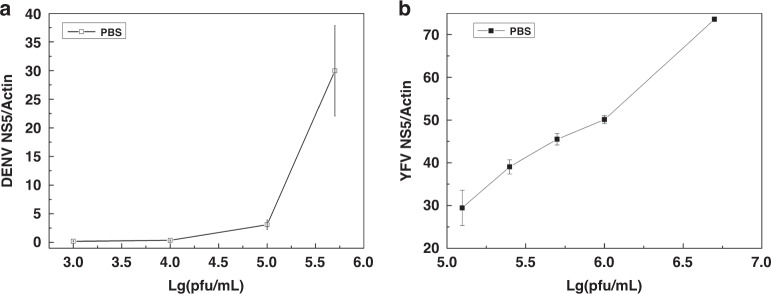
Relative genetic expression of the captured viruses. **a** Relative quantity RT PCR result of the DENV NS5 genetic expression isolated from different DENV concentration samples, with actin as the housekeeper gene, PS microspheres as control group. **b** Relative quantity RT PCR result of the TBEV NS5 genetic expression isolated from different TBEV concentration samples, with actin as the housekeeper gene, PS microspheres as control group

Relative DENV NS5 gene expression was detected by RT‒PCR with actin as the housekeeping gene. As shown in Fig. 7a, the DENV NS5 genetic expression increased along with the DENV concentration and was consistent with the fluorescence intensity. The TBEV we employed was Tick Borne Encephalitis Virus-like particles, which included the Yellow Fever Virus (YFV) genome and Tick-Borne Encephalitis Virus envelope proteins. Here, we detected the relative genetic expression of YFV NS5 to verify that the captured virus was TBEV. The results shown in Fig. 7b indicated that YFV NS5 genetic expression was positively correlated with the virus concentration. The relative genetic expression can reflect the virus concentration and illustrate that the strategy we used for virus sorting and purification was effective and damage-free for subsequent analysis.

## Conclusions

In conclusion, we developed an efficient sorting method based on specific capture of virus by aptamer-modified microspheres and acoustofluidic separation, which is effective and damage-free for subsequent analysis. By this method, contactless and biocompatible separation of two different viruses was accomplished in this work. By modifying the PS microspheres with aptamers, the specification of the captured viruses is labeled by the size of the particles, which corresponds to different radiation forces in the same acoustic field. The acoustofluidic device using TSAW was introduced to complete the sorting of PS microspheres with different diameters. A 15 μm PS microsphere purity of 98.46 ± 2.56% and a 2 μm PS microsphere purity of 99.67 ± 0.57% were obtained after sorting samples containing 23.53 ± 8.77% 15 μm PS microspheres. The combination of aptamer-modified PS microspheres and TSAW devices can prevent matrix interference, reduce detection cost, simplify operation and make it easier to sort viruses. The sorted PS microspheres were detected by LSCM, SEM and RT‒PCR. The observed fluorescence signal and relative genetic expression were positively correlated with the virus concentration, which demonstrates little effect of TSAW sorting on subsequent genetic testing and fluorescence detection. It follows that the sorted virus particles can be employed for subsequent biological detection, such as cell culture, ELISA, Western blotting and PCR tests, which have been used for a long time as tools for the diagnosis of viral diseases in clinical practice. Successful sorting of distinct virus particles from a mixture of biological samples is favorable for improving the detection sensitivity. This work shows a promising method for sample pretreatment in the differential diagnosis of viral diseases.

In addition, multiplexed tests covering multiple infectious agents are especially needed in the case of diseases with indistinct clinical symptoms or where coinfections are possible, such as in fever with acute onset^50^. The sorting method based on specific capture of virus by aptamer-modified microspheres and acoustofluidic separation here can be expanded to multiple virus sorting, and the number of sorted virus species depends on the size differentiation of microspheres by surface acoustic waves. Furthermore, the sorted PS microspheres can be labeled with fluorescent-coupled aptamers for rapid detection of different viruses. Further studies will be carried out to develop a simplified, sensitive, rapid, inexpensive, and less cross-reactive multiplexed detection method for the differential diagnosis of viral diseases.

## Supplementary information

sumpplmentary
