# Supplementary material for: Multiple virus sorting based on aptamer-modified microspheres in a TSAW device
Source: Microsyst Nanoeng. 2023 May 17;9:64. doi: 10.1038/s41378-023-00523-1 (PMC10192341; doi:10.1038/s41378-023-00523-1)
Supplement: Supplementary file 1 — sumpplmentary [file 41378_2023_523_MOESM1_ESM.docx]

**Supplementary Information**

**Multiple virus sorting based on aptamer modified microspheres in TSAW device**

Xianglian Liu^1^, Xuan Chen^1^, Yangchao Dong^2^, Chuanyu Zhang^1^, Xiaoli Qu^1^, Yingfeng Lei^2^, Zhuangde Jiang^1^, Xueyong Wei^1, *^

^1^ State Key Laboratory for Manufacturing Systems Engineering, Xi’an Jiaotong University, Xi’an, 710049, P.R. China

^2^ Department of Microbiology, the Fourth Military Medical University, Xi’an, 710032, P.R. China

**Corresponding author’s Email: seanwei@mail.xjtu.edu.cn**

**TableS1.** The sequences of aptamers and primers employed in the experiments.

| **Name** | **Sequence(5’→3’)** |
| --- | --- |
| **DENV capture aptamer** | NH_2_/-CCCGCACCGGGCAGGACGTCCGGGGTCCTCGGGGGGCGGG |
| **DENV mark aptamer** | GCACCGGGCAGGACGTCCGGGGTCCTCGGGGGGC-/6-FAM |
| **TBEV capture aptamer** | NH2/-ATCCGTCACACCTGCTCTACATCACATCTGACTGAGTCCATGGCGCTCTGTGTGTGGTGTTGGCTCCCGTAT |
| **TBEV mark aptamer** | ATACGGGAGCCAACACCACCGCAGGAGTCCATCAGGGGTTGGCAGTCAGCGCTCAGAGCAGGTGTGACGGAT-/Texas Red |
| **DENV NS5 Forward Primer** | GGTTTTGGGAGCTGGTTGAC |
| **DENV NS5 Reverse Primer** | ACTCTAAGAAGCGTGCTCCA |
| **YFV NS5 Forward Primer** | GTTCCACAAGGACGCACAAC |
| **YFV NS5 Reverse Primer** | TGATCCGCACAGCTTGTCTT |
| **Actin Forward Primer** | CATGTACGTTGCTATCCAGGC |
| **Actin Reverse Primer** | CTCCTTAATGTCACGCACGAT |


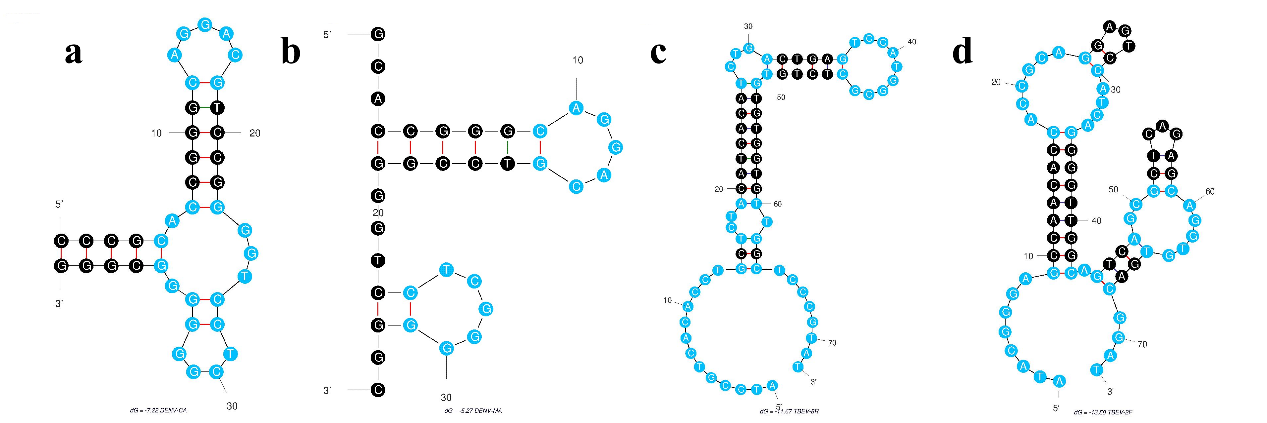


**Fig S1.** The secondary structures of the DENV capture aptamer and the DENV mark aptamer analyzed by Mfold web platform. (a) DENV capture aptamer; (b) DENV mark aptamer; (c) TBEV capture aptamer; (d) TBEV mark aptamer.


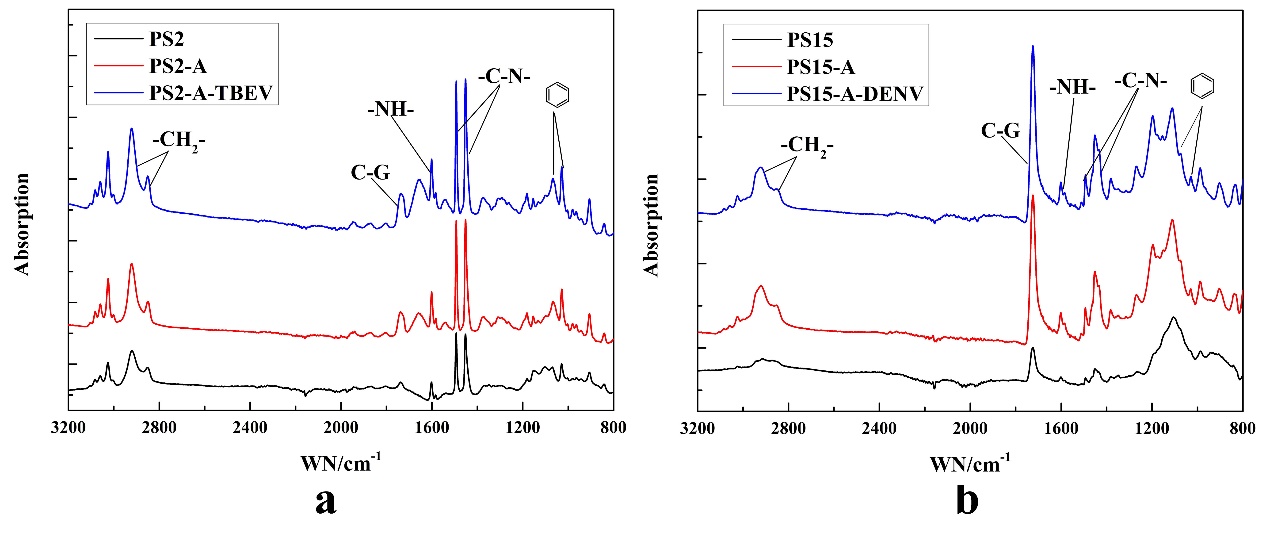


**Fig S2.** The FTIR spectrogram of PS microspheres. (a) The FTIR spectrogram of 2μm PS microspheres (PS2), aptamer conjugated 2μm PS microspheres (PS2-A) and the complexes of PS microsphere-Aptamer-TBEV(PS2-A-TBEV); (b) The FTIR spectrogram of 15μm PS microspheres (PS15), aptamer conjugated 15μm PS microspheres (PS15-A) and the complexes of PS microsphere-Aptamer-DENV(PS2-A-DENV).
